# Essay on Some Diseases of the Heart

**Published:** 1814-03

**Authors:** Chevalier Pelletan


*For the Medical and Physical Journal.*

### *Essay on some Diseases of the Heart; by the* CHEVALIER PELLETAN.

### *State of Inertia or Palsy of the four Parts of the Heart.*

A MAN thirty-six years of age, an Irishman by birth, five feet seven or eight inches high, was of an athletic frame, and a countenance strongly colored. His character and his passions were in exact relation to his organization. Watching and play, women and good cheer, hardly sufficed for his prodigious activity.

Carried away by the revolutionary torrent, he had received an important mission to Saint Domingo, about the end of the reign of Louis XVI. The system which followed extended almost immediately to the place of his mission, and caused him the greatest difficulties and calamities. Brought back by authority to France, he was three times cited before the revolutionary tribunal, and each time owed his safety solely to the vigour of his eloquence; his commanding manner, and the power of his voice, rebuffed the sanguinary cowardice of this tribunal.

A more favorable horizon appearing in France, our man attempted to indemnify himself for his losses. Numerous and urgent wants stimulated his passions, so that he merely changed the nature of his torments by want of success with a government, whose character was moderation, and whose interest it was to remove violent people. He at last, however, obtained a pension of 12,000 francs, which, being given without good reasons, was taken away from him, on the death of the patron who had procured it. This last misfortune was fatal. He has told me an hundred times, that on hearing the news of his loss, he immediately felt a dreadful weight in the chest. His respiration became fatiguing, and the pulsations of his heart assumed an irregularity, which had no interruption during the two years and an half that he survived his misfortune.

Being reduced during this interval to betake himself to the profession of physic, which he had formerly embraced, he exercised it with as little honor as profit. Want, and the torture of finding resources, which were neither delicate nor productive, disputed for the reliques of an existence, which was devoted to despair, and a prostration of moral and physical power, which formed an astonishing contrast with the former vigour of this individual.

The habitual character of his pulse was quickness, weakness, and irregularity; the pulsations of the heart were felt only in the usual place. The face was rather violet-colored than florid. Oppression was the general feeling produced by respiration. The appetite kept up, although the patient ate indolently. The emaciation, which was never extreme, seemed to depend less on physical than moral causes. In this state the patient dragged out his existence for more than two years. He habitually used antispasmodics, and sometimes had recourse to bleeding, which did not relieve him. At last the respiration became more difficult, and the spasms of the heart insupportable: the patient was compelled to keep his chamber, and afterwards his bed; the feet and legs swelled; no position of the body relieved his respiration; a spitting of blood took place, which relieved the patient at first, and induced him to apply leeches to the breast. This symptom soon recurred without the former good effect; but the irregular pulsation of the heart produced a distress approaching to death. At last this man sunk on the 23d May, 1806, two years and five months after the fatal event which had determined the existence of his disease.

On examining the body, the following appearances presented themselves:

Externally, bluish and reddish ecchymoses along the right side of the face and the body; little œdema of the lower extremities; and traces of bloody mucus from the nose and mouth.

The chest being struck on the right side did not resound, and had a slight œdema. On the left side the sound was full and natural.

On opening the chest we found that its right cavity contained about a pint and an half of water; and the left only half a pint. The lungs were sound, not adherent to the pleura, and of natural color; the inferior lobes on each side were bluish, increased in bulk, and their vessels unusually full full of blood. These appearances were most strongly marked in the right lung, which was the most weighty, though the admission of air had not been prevented.

The heart, covered by its pericardium, was in its natural situation. The pericardium of a natural and healthy thickness, without any adhesion to the surface of the heart. It contained about four ounces of serosity.

The right cavities of the heart and the venæ cavæ were more dilated than common, without being absolutely aneurismal. They contained a moderate quantity of blood in the form of coagula. The pulmonary artery and veins were perfectly healthy.

The left auricle, ventricle, and the aorta, were healthy. The auricle and ventricle contained as much blood as those of the right side.

But what was very evident, and which I had never before witnessed, was the state of flaccidity of the whole substance of the heart. The parietes of the cavities fell together, and the flesh of this organ might be compared to the pale and shrunken muscles of an old woman: there was an astonishing contrast between the flesh of the heart and that of the other muscles of the body. I do not hesitate to believe that the heart was struck with a kind of palsy, and that its feeble, precipitate, and irregular contraction was the effect of an action not sufficient to disembarrass it of the blood which flowed into its different cavities.

It is very surprising that such an organic derangement had not occasioned an unnatural dilatation of all the cavities of the heart; that it was not followed by hardness of the lungs, dropsy of the pleura, of the pericardium, and even of the whole body; for it must be allowed that the water we found effused in the cavity of the chest, was not in proportion to the disease of the heart, and even not sufficient to cause death.

I believe, and every one will agree with me, that death resulted from the total palsy of the heart.

Let us remember what the patient had said during the whole course of his complaint: he had felt at the instant the loss of his pension was announced, an enormous weight in the chest. Patients struck down with a violent affection, paint what passes in them with sufficient truth. One feels a weight and sinking which represent the inertia of the heart, as in the above case; another says, that he has the heart tied up or constricted, and very well expresses the reaction of the pericardium on the heart, when blood with difficulty penetrates the vessels of the lungs; some say they feel a weakness accompanied with pulling or drawing on the heart, in

 the the state of inanition brought on by an abstinence not alleviated by drinks, &c.

The opening of the abdomen exhibited a kind of disease which grief and melancholy very often produce; the biliary vesicle was much dilated with a blackish bile, and contained as many as twenty biliary concretions of different sizes, the largest being as large as a filbert. The liver was perfectly healthy.

It is a common opinion that gall stones are found in the vesicle of persons who commit suicide. I have ascertained this fact a great number of times, in subjects who had been induced to self murder by long distress; but never in those who had committed it in consequence of sudden grief and despair, such as takes place after losses in gaming, or from disappointed love. The languor in which this man lived, more than two years, explains the formation of his gall stones.

No other organ was disordered. The functions of the brain had not been disturbed during life; nor did we find any thing wrong in this organ, or its membranes.

The man, whose history we have related, might have been affected with an inflammation of the heart during the active part of his life, and thence had a disposition to be violently affected by such a cause as produced his disease. We shall now see an example of this inflammation of the heart, brought on by the effect of violent passions and emotions.

*Inflammation of the Heart and other Organs of the Thorax, and of the Abdomen, brought on by violent Passions, and excesses in Eating and Drinking.*

All Europe has resounded with the oratorical talents as well as the impetuous character of Mirabeau. I am far from wishing to imprint a stain on his memory; but it is necessary I should say a word of his private conduct and his character, in order to point out their influence on the disease which proved fatal to him.

Cabanis, a physician of talents, was his intimate friend. He attended him in his last illness. He has left us a history of it, which does as much honor to the sagacity of the author, as to the tenderness of heart with which he speaks of his friend. He has, above all, a desire to exculpate him from the various charges made against his character; and yet, compelled by the force of truth, he accuses Mirabeau beyond what is necessary, to preserve him the reputation of a man whose passions were increased to an inordinate degree, and whose immorality led him to sport with all opinions, and to defend them all by turns, when the interest of the moment, the the desire of shining, or any other motive, induced him to attempt to persuade to what he wished to obtain.

The result of such a character was, that Mirabeau possessed a factitious or deceitful eloquence, and sensations at command. He supplied the place of that vehemence, inspired by a strong conviction of truth, by a force of pulmonary organ, which had no example, and by contortions of the muscles of the face and whole body, which rendered him hideous to behold at the tribune; at the same time that, according to my own feelings, he never carried less conviction into the mind of his auditors, than when he most attempted to be eloquent.

The abuse he made of the pleasures of the table was but too often his Apollo; and this regimen, which he was accustomed to, rendered him most amorous of women, to whom he devoted all the time he could spare from the necessity of appearing in public.

Mirabeau, being named president of the constituent assembly on the 29th January, 1791, entered on a career in which he abused his powers and his resources beyond all measure. *It was necessary*, says his panegyrist, *to employ very active and very speedy methods of putting him in a state to terminate his fortnight. He had oppressions, diaphragmatic contractions, pains at the superior orifice of the stomach, which terminated by bilious dejections, either spontaneous, or provoked by means of the waters of Sedlitz. Baths sometimes diminished them.*

One day, on stooping precipitately, he experienced the most severe agonies in the precordium, which almost made him faint. Perhaps this was the first indication of that disease of the heart which was discovered after his death; but Cabanis took little notice of it, *knowing*, says he, *how obscure these diseases are, and how problematical their existence is, even when announced by constant, palpable, and unequivocal symptoms, and how hazardous is the treatment of them. However, the habit of committing imprudences increased every day, and these approached more and more near to each other.*

At this epoch, Mirabeau had a first attack of colic, which was relieved by baths, and terminated by bilious evacuations. A second assumed a very marked spasmodic character, and terminated in requiring an emetic and some saline purgatives. Immediately after this colic, the sick man committed an excess at table, in a nocturnal repast; and he did not stop at this fault, already sufficiently great. It was on the night between Saturday and Sunday of the 27th March; he had a colic, attended with inexpressible agonies; and in spite of all the representations that were made to him, he insisted on going to the assembly, where they were the next day to discuss the affair of the mines; and he mounted the tribune five times, to obtain by his eloquence a decision in favor of his opinion. It was, according to the expression of Cabanis, the song of the swan.

On returning home, Mirabeau experienced the most violent pains; they had seized the great curvature of the intestine colon; they extended with violence under the sternum, and reached all the external and internal dependencies of the chest, diaphragm, precordial region, mediastinum, breasts, and clavicles. Every where they produced the sensation of a talon of iron, which violently tore the sensible parts.

The anxieties were very great; the respiration was so impeded as to threaten instant suffocation; the countenance was bloated from the arrest of the blood in the lungs; the pulse intermittent and convulsive; the extremities cold; and the patient making vain efforts to restrain the groans which distress extorted from him. His physiognomy exhibited the impression of fatal disease,—never did a patient appear so decidedly struck with death.

In this state of universal inflammation, he had a bleeding from the foot, large blisters were applied to the full part of the leg, and strong sinapisms covered over all the lower part these extremities. The pulse became more regular, the respiration more free, and he had a copious sweat. Wednesday morning the symptoms reappeared: the pulse was quick and hard; the head heavy and painful; the arterial, precordial, and diaphragmatic spasms returned. The treatment was restricted to simple diluents; but very soon the pains increased, the spasms extended over the whole chest, the right shoulder-blade, clavicle, and the region of the diaphragm. The pulse became intermittent and convulsive. The applications of cantharides and mustard were repeated, but became insupportable from the pain, and were removed. The countenance turned yellow, the tongue became coated; and this decided the indication to evacuate the bile by the Sedlitz water and whey. The commotion in the system abated, but in the evening increased with greater or less violence. About three o'clock in the morning, the pains began to display the same *ferocity*; the pulse had the character of intermittence and convulsion; the suffocation, the spasms, and all the frightful train which at first accompanied them, returned with rapid strides. The bleeding in the foot was repeated, and the application of sinapisms containing cantharides; the blisters in the legs were irritated afresh, and new ones applied to the thighs; finally, a pill containing six grains of musk was was given every half hour, till the patient had taken from thirty to forty grains.

This last accession continued a long time, and left the patient with the aspect of death.

It was thought that the alternatives of relief and paroxysm denoted the existence of an intermitting malignant fever, and cinchona was resorted to; but the difficulties were increased by it, and the patient vomited after the third dose. The respiration now became worse every instant, together with spasms and the most insupportable pains, and the most frightful physiognomy. The result of a consultation with M. Antoine Petit, was the application of more blisters to the arms, and the administration of half a grain of camphor, every half hour. At the end of six hours, the wounds of the blisters were cupped and washed with volatile alkali, which was repeated a number of times. The pains were increased to a degree impossible to be expressed. The patient, in agony, begged for opium; and when he could no longer be heard, he collected strength enough to write, *sleep*. It was necessary to appear to yield to his entreaties; and while a calming potion was preparing, he, on the sixth day of his disease, expired.

The picture that I have sketched of the symptoms of the disease of Mirabeau is extracted, word for word, from the relation of his friend Cabanis. I did not myself see the patient, but hearing every day a particular account of his situation, I could not avoid recognizing the existence of a most violent inflammatory disease, and which ought to have been combated by the flow of rivers of blood. I know not what idea his friend had formed; what system of humors, fixed in the affected parts, he had adopted, on the pretext that the patient had been subject to ophthalmia and acute pains of the bowels. He, however, states in many parts of his recital, that he attributed the ophthalmia to an old venereal taint, for which he was on the point of administering a methodical treatment; and the affections of the bowels to the excesses of every kind in which Mirabeau indulged. But it must be observed, that in the whole relation Cabanis takes as much pains to conceal from himself the inflammatory disease, of which he has described the most characteristic symptoms, as he does to praise and extol his hero, while he depicts all the vices of his character and conduct.

The fatal effect of the prejudice of the physician was, that fresh fuel was heaped on the flame. The body was covered with blisters and vesications to a degree that no case in medicine ever indicated. Saline purgatives, camphor, musk, and cinchona, were administered internally. It is well to observe, observe, that what kept up the delusion of the physician, was the momentary relief which took place after the first application of the external irritants. There is no doubt that these violent factitious irritations might for a short time suspend the principal irritation; but this increased afterwards with a violence proportioned to the addition of irritating causes; and if we read the account with attention, we shall be convinced that the apparent success was illusory, and perceived by Cabanis alone. It must be said, in his justification, that he felt too lively an interest in the health of his friend, whom he loved with enthusiasm, to possess that tranquillity of mind, without which a physician cannot study, observe, or recognize the truth.

I was desired to examine the body of the deceased; but here also I may admit Cabanis to speak, for he gives a faithful recital of what was observed.

"The stomach, the duodenum, a great part of the liver, the right kidney, the diaphragm, and the pericardium, exhibited marks of inflammation, or rather, in my opinion, of congestion of blood. The pericardium contained a considerable quantity of thick, yellowish, opaque matter. Lymphatic coagulations covered the whole external surface of the heart, except the point. The cavity of the chest contained a little water.

"Certainly the state of the heart and the effusions in which this organ floated, may be considered mortal; but I believe that death was immediately determined, by the affection of the diaphragm; and I have ever attributed this affection, as well as that of the heart, to the rheumatic, gouty, wandering humor, which we from the beginning had considered as the cause."

Cabanis might regard the pretended gouty humor as the cause of the inflammatory disease; but it did not the less possess this inflammatory character in the very highest degree. All the organs named were inflamed; one would have said that a poison had corroded the stomach and duodenum; and the intestines were empty, as happens in such a case. The heart and pericardium were red with inflammation; independently of the mass of lymphatic concretions, which constantly characterise the inflammation of this organ, that of the liver, right kidney, and surrounding cellular membrane, was much less; but it is certain that the state of the diaphragm alone would have explained the violence of the pains to which the patient was a prey. The right side of this muscle was contracted, just as if it had preserved, after death, the state of most powerful action.

It will be admitted that, instead of having recourse to an occult cause to explain so violent and extensive an inflammation, the origin of the disease may be imputed to the life which Mirabeau had led previous to the invasion of his last illness, to his excesses of every kind, to that even which he committed at the tribune of the assembly, when he mounted five times in one sitting, and when he was already gravely attacked by the disease to which he fell a victim.

The conclusion of Cabanis is well worthy of the warmth of imagination which dictated his history. *I declare candidly*, says he, *that on seeing the same series of symptoms again, I should form the same judgment, and should employ the same methods of cure.* For my part, I conclude that Cabanis was the Seïde of his medicine, and Mirabeau the victim of the fanatic enthusiasm of his physician.

I cannot reproach myself for having given to the history of the disease of Mirabeau the high degree of importance it has seemed to me to possess. It is one of the most important and instructive facts that any disease of this nature can present. I had formed a design, at the very period of the event, to submit the relation of Cabanis to a critical examination, proportioned to the importance of its object; but considerations of respect towards so excellent a character arrested my pen. Now that he has paid the tribute to nature, and that Mirabeau is no longer an object of revolutionary admiration, I fulfil the obligation I had contracted, of employing for general instruction all the interesting facts, of which I can render an exact and faithful account.

We may draw a curious comparison between Mirabeau and the subject of the first observation. The latter, possessed of physical powers far greater than those of Mirabeau, had nothing like his genius and the force of his character; and he allowed himself to be beat down by an event, against which Mirabeau would easily have found resources. I have already remarked, that this man might have been attacked with inflammation of the same kind as Mirabeau, in the circumstances when he had to defend his life by the power of his eloquence; but he sunk under a palsy of the heart, the effect of profound grief. On the contrary, Mirabeau fell a victim to the violence of his temperament, of his passions, and of the abuse he made of all his faculties.

